# Mental health and quality of life in firefighters working on the scene in South Korea: Focused on the capital area and the ground pro‐motion area

**DOI:** 10.1002/brb3.1559

**Published:** 2020-03-03

**Authors:** Woo‐Hyuk Jang, Da‐Som Kim, Hye‐Won Park, Ji‐Hoon Kim

**Affiliations:** ^1^ Department of Occupational Therapy College of Health Science Kangwon National University Samcheok‐si Korea

**Keywords:** mental health, occupational stress, psychological well‐being, quality of life, working area

## Abstract

**Introduction:**

Today, firefighters' work areas are expanding into various fields. The aim of the study was to investigate the effect of mental health on quality of life between firefighters in the capital area and in the growth promotion area.

**Method:**

A survey was conducted with 206 firefighters including 110 firefighters in the capital area and 96 firefighters in the growth promotion area through a questionnaire.

**Result:**

The differences in mental health based on working areas between firefighter groups have been identified. In addition, among the factors affecting the quality of life among firefighter, the factor most closely associated with quality of life was “sociopsychological health stress,” followed by “occupational stress,” “depression,” and “working area.”

**Conclusion:**

There is a need for research into mental health interventions appropriate for regional characteristics of each region regarding the implementation of mental health management by working area.

## Graphical abstract

Effect of mental health on quality of life in firefighters

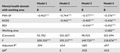

## 1 INTRODUCTION

Today, firefighters' work areas are expanding into various fields (Chae, Woo, & Ko, 2012). With the development of society, work of firefighters is not limited to fire‐related activities such as fire prevention, fire precaution, and fire suppression but has been diversified into various areas, and they are responsible for all safety accidents occurring across the country, including rescue and emergency services (Chae et al., 2012). According to the statistics of the National Fire Agency, the work of firefighter has been steadily increasing over the past decade from 2008 to 2017, mainly in the areas of rescue, first aid, and life safety (National Fire Agency 119, 2018). Moreover, they should always be ready to immediately respond to urgent situations such as disasters, accidents, and emergencies that may occur at any time (Choo, Park, & Kang, 2011). In stressful situations such as the intensity of shocks at the scenes of accidents through firefighting activities and threats to life in dangerous situations (Kim, Kim, & Kim, 2006), the psychological burden and stress of firefighter working at the scene of an accident or emergency are continuously increasing (Choo et al., 2011).

In this situation, many studies have been conducted on the mental health of firefighters (Choi, Kim, & Cho, 2009; Ha et al., 2008; Ha et al., 2008; Kang, 2009; Kang, 2009; Kim, 2002; Moon, 2011; Saijo, Ueno, & Hashimoto, 2008; Yoon & Kim, 2014). According to Ha et al. (2008) and Kang (2009), the level of perceived stress was found to be higher among firefighters than among the general occupation groups (Ha et al., 2008; Kang, 2009). In addition, regarding the stress level among firefighters, it has been reported that 90% of study participants were found to work under a serious level of stress (Choi et al., 2009). In particular, large and small stressful situations in the workplace cause firefighter to experience exhaustion, and such exhaustion lowers the level of job satisfaction and hinders effective work performance (Kim, 2002). A study of Japanese firefighters reported that the burden of various tasks negatively affects the mental health of firefighters (Saijo et al., 2008). In Korea, firefighters are exposed to a high level of occupational stress since their occupation requires working 2 shifts per day on a 24/7 basis and activities to cope with the experience of traumatic events (Moon, 2011). In a related previous study, firefighters whose occupational stress level was in the top 50% were 43.7% of all the study participants (Yoon & Kim, 2014). It has also been reported that since this occupational stress negatively affects mental health, the management of occupational stress should be considered as a crucial element for mental health among firefighters in Korean society (Ha et al., 2008).

On the other hand, Korean society has very high regional imbalance, and this imbalance is getting worse over time (Lee et al., 2013). The functions in all areas including politics, economy, health and education, society, and culture are concentrated in the capital area (Lee et al., 2013). Cho (2000) noted that these subareas are likely to result in regional disparities in the quality of life (Cho, 2000). In addition, regional differences are also shown by the study results of differences in life satisfaction between the capital area and noncapital areas as well as the differences in mental health resulting from occupational stress (Lee, 2017; Um & Kim, 2013). Lee (2017) reported that while the level of health satisfaction was higher in the capital region, the level of job satisfaction including the satisfaction level for the residential environment and leisure satisfaction was higher in other regions (Lee, 2017). In addition, based on the study results that there are differences in mental health among local government public servants according to the workplace, Um and Kim (2013) suggested that there are likely to be regional differences between the capital region and the noncapital regions and that it is necessary to expand the research on occupational stress among public servants to diverse regions rather than limiting it to a specific region (Um & Kim, 2013). According to a study by Back and Lee (2006), there is a regional difference in the fire service force that supports the work of firefighters, because the criteria for the fire service force are specified according to the type of village based on the jurisdictional area and the jurisdictional population. However, it is reported that the actual operation and organization are delegated to local governments of cities and provinces (Back & Lee, 2006).

As mentioned above, many studies have been conducted on the mental health of firefighters, including their occupational stress. However, although some previous studies considered the mental health of firefighters in specific regions, there have been few studies of regional differences in mental health and the quality of life related to the regional differences.

Therefore, this study aims to investigate the effect of mental health on the quality of life between firefighters working in the capital area and in growth promotion areas1Areas with a poor living environment and a low development level which need special consideration of the central and local governments for the establishment of the infrastructure etc. Among cities and counties in Korea, they are jointly designated and announced by the ministers of the Ministry of the Interior and Safety and the Ministry of Land, Infrastructure and Transport through the comprehensive evaluation of several criteria such as the annual average population change rate and the income level. among noncapital areas.

## 2 METHODS

### 2.1 Participants and the study period

A survey was conducted for 3 months from September to November 2018. The participants of this study were a total of 206 people, and they consisted of 110 firefighters in the capital area and 96 firefighters in the growth promotion area. The questionnaire survey in the capital area was conducted among firefighters working in sixteen 119 safety centers located in Seoul, Gyeonggi Province, and Incheon, and the survey in the growth promotion area was conducted among firefighters from nineteen 119 safety centers located in Gangwon and Gyeongsang Provinces among the regions presented in Appendix. The survey participants were firefighters operating on the scene who worked at 119 safety centers (shift work system), and their consent was obtained by contacting them in advance. The survey was conducted using self‐administered questionnaires among the participants who voluntarily agreed to participate and signed informed consent forms after the researcher personally visited 119 safety centers and fully explained the purpose of the study and the contents of the questionnaire.

### 2.2 Measures

#### 2.2.1 General characteristics

General characteristics included a total of ten variables such as sex, age, education, religion, marital status, task, rank, working period, motivation for becoming firefighter, and desired period of service.

#### 2.2.2 Domains of mental health

##### Beck Depression Inventory (BDI)

To assess the degree of depression, a Korean version of the BDI developed by Beck, Ward, Mendelson, Mock, and Erbaugh (1961) were used. The Korean version of BDI was developed by Lee and Song (1991) by translating and modifying the original version created by Beck in 1961. This assessment tool is used to measure the change in the depression index, and Cronbach's coefficient of reliability is *α* = 0.86 (Eom, 2013). It is a self‐report instrument which consists of a total of 21 items encompassing Cognitive, Affective, Motivational, and Physiological domains, and each item is rated on a Likert‐type 4‐point scale with the score range of 0–3 points (Cho & Kim, 2002). The total scores range from 0 to 63 points, and higher scores indicate higher levels of depression. With respect to the interpretation of scores, the BDI scores are considered to indicate the normal state or degree of depression: The score of 0–9 points indicates “the normal state”; 10–15 points “mild depression”; 16–23 points “moderate depression”; and 24–63 points “severe depression” (Beck, 1976).

##### Psychosocial Well‐being Index—Short Form (PWI‐SF)

PWI‐SF was used to assess sociopsychological health stress. This tool is a short form of the Psychosocial Well‐being Index (PWI), which consists of 45 items, and PWI‐SF was created through the modification of the items and scale of the original version in two stages to make it suitable for the Korean situation in 2000. Cronbach's reliability coefficient was *α* = 0.832 (Kim & Lee, 2013). PWI‐SF consists of a total of 18 items, and each item is rated on a Likert‐type 4‐point scale with the score range of 0–3 points, and the total scores range from 0 to 54 points. Higher scores indicate the more severe levels of sociopsychological stress. Regarding the interpretation of scores, the score of 0–8 points is considered to indicate “the health group”; 9–26 points “the potential stress group”; and 27 points or above “the high‐risk group.” In the case of the items where higher scores indicate lower levels of social psychological health stress, they were reverse scored by recoding. Reverse scored items are Items 1, 5, 6, 8, 9, 10, 11, 12, 14, 17, and 18, and they are positive statement items (Chang, 2000).

##### Korean occupational stress scale (KOSS)

In order to assess occupational stress, KOSS, which was developed through the development and standardization of a Korean occupational stress measurement instrument in 2003, was used. It is a measure to assess the occupational stress level of workers and identify the risk factors of individuals and work affecting occupational stress. Cronbach's reliability coefficient was *α* = 0.844 (Yoo, Choi, Song, & Lee, 2011). This scale consists of a total of 43 items, and each item is rated on a Likert‐type 4‐point scale. The scores are converted into scores out of 100 points. The total score obtained by adding the scores of all domains is divided by the number of areas. A higher score indicates a higher level of stress. The subareas of KOSS include physical environment, job demand, insufficient job control, interpersonal conflict, job insecurity, organizational system, lack of reward, and occupational climate. In the case of items where higher scores indicate lower levels of occupational stress, they were reverse scored by recoding. The reverse scored items are Items 1, 9, 12, 14, 15, 16, 17, 18, 19, 20, 21, 22, 24, 27, 28, 29, 30, 31, 32, 33, and 35, and they are positive statement items (Chang et al., 2005).

##### World Health Organization Quality of Life Scale (WHOQOL‐BREF)

To determine the quality of life index, we used the Korean version of the WHOQOL‐BREF developed by Min, Lee, Kim, Suh, and Kim (2000). This is a self‐administered questionnaire to assess the quality of life (Jeon, Jeon, Yi, & Cynn, 2014). The reliability of this instrument was Cronbach's *α* = 0.92. It consists of a total of 26 items, and each item is rated on a Likert‐type 4‐point scale with the score range of 1–4 points. The total scores range from 26 to 130 points, and higher scores indicate higher levels of the quality of life (Jeon et al., 2014). The subdomains of WHOQOL‐BREF are the overall domain, physical domain, psychological domain, social domain, and environment domain, and the items reverse scored by recoding were Items 3, 4, and 26, and they are negative statement items (Kim, Lee, & Park, 2011).

### 2.3 Data analysis

The data collected in this study were analyzed using the SPSS 18.0 program as follows. Normality is satisfied with a large number of subjects. Frequency analysis was conducted to identify the general characteristics of the participants. In addition, the chi‐square test was used to compare general characteristics between two groups for the homogeneity test. The independent sample *t* test was conducted to examine differences in mental health domains and the quality of life between groups. Moreover, Pearson correlation analysis was performed to examine the correlations between general characteristics and mental health domains in each group and between the quality of life index and mental health domains. In addition, multiple regression analysis was performed for a causality test with the mental health domains and quality of life scale. All statistical significance levels were set at 0.05.

## 3 RESULTS

### 3.1 General characteristics

The participants of this study were firefighters in charge of on‐scene firefighting and emergency services (shift work system) working at the 119 safety centers. A total of 206 participants, including 110 firefighters in the capital area and 96 firefighters in the growth promotion area, were included in this study. The general characteristics of participants are shown in Table 1. A test for homogeneity for the general characteristics of the capital area and the growth promotion area groups was performed, and as a result, there were no statistically significant differences, showing the homogeneity of the two groups (Table 1).

**Table 1:** General characteristics of the participants

	Characteristics	Capital area	Growth promotion area	*χ* ^2^	*p*
		*n* (%)	*n* (%)		
Sex	Male	98 (89.1)	92 (95.8)	3.25	.071
	Female	12 (10.9)	4 (4.2)		
Age (year)	20–29	21 (19.1)	26 (27.1)	5.25	.154
	30–39	45 (40.9)	26 (27.1)		
	40–49	35 (31.8)	32 (33.3)		
	50	9 (8.2)	12 (12.5)		
Education	High school	29 (26.4)	22 (22.9)	1.24	.536
	University	80 (72.7)	74 (77.1)		
	Graduate school	1 (0.9)	0 (0.0)		
Religion	Yes	27 (24.5)	27 (28.1)	0.357	.837
	No	82 (74.5)	68 (70.8)		
	etc.	1 (0.9)	1 (1.0)		
Marital status	Yes	39 (35.5)	32 (33.3)	0.102	.749
	No	71 (64.5)	64 (66.7)		
Task	Rescue& first aid	30 (27.3)	37 (38.5)	3.19	.203
	Firefighting drive	32 (29.1)	26 (27.1)		
	Fire suppression	48 (43.6)	33 (34.4)		
Rank	Firefighter	34 (30.9)	27 (28.1)	3.29	.510
	Fire engineer	30 (27.3)	25 (26.0)		
	Fire lieutenant	27 (24.5)	19 (19.8)		
	Fire captain	19 (17.3)	24 (25.0)		
	Fire marshall	0 (0.0)	1 (1.0)		
Working Period (year)	<1	12 (10.9)	16 (16.7)	9.730	.373
	1–3	17 (15.5)	15 (15.6)		
	3–6	11 (10.0)	6 (6.3)		
	6–9	13 (11.8)	15 (15.6)		
	9–12	20 (18.2)	6 (6.3)		
	12–15	5 (4.5)	4 (4.2)		
	15–18	7 (6.4)	6 (6.3)		
	18–21	8 (7.3)	8 (8.3)		
	21–24	6 (5.5)	9 (9.4)		
	≥24	11 (10.0)	11 (11.5)		
Motivation for becoming Firemen	Stability	25 (22.7)	30 (31.3)	4.591	.332
	Aptitude, Interest	48 (43.6)	42 (43.8)		
	Recommendation	16 (14.5)	8 (8.3)		
	Wage	0 (0.0)	1 (1.0)		
	etc.	21 (19.1)	15 (15.6)		
The desired period of service	As long as possible	73 (66.4)	65 (67.7)	2.494	.287
	Until necessary period	37 (33.6)	29 (30.2)		
	etc.	0 (0.0)	2 (2.1)		

### 3.2 Verification of differences in mental health domains and quality of life according to the working area (the capital area and growth promotion area)

Prior to the analysis of collected data, participants were divided into the capital area and growth promotion area groups. Then, verification of differences between the two groups was conducted using the total score of each of the Beck Depression Inventory (BDI), the Psychosocial Well‐being Index—Short Form (PWI‐SF), the Korean occupational stress scale (KOSS), and the World Health Organization Quality of Life Scale (WHOQOL‐BREF). In addition, verification of differences in 8 subareas of KOSS between two groups was also conducted. As a result, there were significant differences between groups in all mental health domains except WHOQOL‐BREF. In addition, there were significant differences between two groups in all the subareas of KOSS except job demand and job insecurity (Table 2).

**Table 2:** Mental health domain of participation by area

Mental health domain with WHOQOL‐BREF	Capital area	Growth promotion area	*t*	*p*
	*M* ± *SD*	*M* ± *SD*		
BDI	6.60 ± 7.03	4.62 ± 5.13	2.322	.021*
PWI‐SF	19.66 ± 9.64	17.00 ± 8.38	2.099	.037*
KOSS	28.15 ± 7.93	24.94 ± 6.77	3.094	.002**
WHOQOL‐BREF	74.50 ± 12.43	75.64 ± 10.19	−0.726	.468
Subareas of KOSS				
Physical environment	38.74 ± 13.28	33.75 ± 10.81	2.930	.004**
Job demand	23.90 ± 10.72	24.55 ± 9.66	−0.454	.651
Insufficient job control	34.40 ± 9.57	31.40 ± 11.55	2.039	.043*
Interpersonal conflict	23.34 ± 9.85	19.14 ± 9.79	3.058	.003**
Job insecurity	25.07 ± 8.57	22.75 ± 10.74	1.726	.086
Organizational system	28.35 ± 12.27	24.58 ± 10.60	2.367	.019*
Lack of reward	25.11 ± 10.06	21.96 ± 9.34	2.317	.022*
Occupational climate	26.67 ± 12.79	22.28 ± 11.67	2.558	.011*

Abbreviations: BDI, Beck Depression Inventory; KOSS, Korean occupational stress scale; PWI, Psychosocial Well‐being Index—Short Form; WHOQOL‐BREF, World Health Organization Quality of Life Scale.

* *p *< .05.

** *p *< .01.

### 3.3 Correlations between mental health domains, quality of life, and working area

The correlation analysis among the domains of mental health which showed a significant difference between groups, quality of life, and working area was performed, and the results showed that all the domains of mental health were correlated with each other, and all of them were also correlated with quality of life. Among them, PWI‐SF showed the highest correlation with quality of life, followed by BDI and KOSS. In addition, working area had no correlation with quality of life, but it was correlated with all the other mental health domains (Table 3).

**Table 3:** Correlation analysis of quality of life and mental health domain

	1	2	3	4	5	6	7	8	9	10	11
1	1										
2	−0.667**	1									
3	−0.772**	0.650**	1								
4	−0.648**	0.478**	0.576**	1							
5	−0.385**	0.290**	0.325**	0.671**	1						
6	−0.266**	0.186**	0.278**	0.599**	0.214**	1					
7	−0.374**	0.195**	0.314**	0.606**	0.245**	0.425**	1				
8	−0.579**	0.455**	0.516**	0.755**	0.427**	0.336**	0.309**	1			
9	−0.612**	0.519**	0.546**	0.819**	0.448**	0.433**	0.424**	0.680**	1		
10	−0.485**	0.392**	0.465**	0.759**	0.406**	0.399**	0.417**	0.504**	0.618**	1	
11	0.050	−0.158*	−0.145*	−0.212**	−0.201**	−0.141*	−0.209**	−0.162*	−0.160*	−0.176*	1

Note

1, World Health Organization Quality of Life Scale; 2, Beck Depression Inventory; 3, Psychosocial Well‐being Index—Short Form; 4, Korean occupational stress scale; 5, Physical environmen (Subareas of KOSS); 6, Insufficient job control (Subareas of KOSS); 7, Interpersonal conflict (Subareas of KOSS); 8, Organizational system (Subareas of KOSS); 9, Lack of reward (Subareas of KOSS); 10, Occupational climate (Subareas of KOSS); 11, Working area.

* *p *< .05.

** *p *< .01.

### 3.4 The influences of mental health domains and working area on quality of life

Multiple regression analysis was conducted to examine the influences of the mental health domains (PWI‐SF, KOSS, BDI) which showed significant differences between the capital area and growth promotion area groups and working area on quality of life. To avoid the problem of multicollinearity, the subareas of KOSS were excluded from analysis items before regression analysis was performed. In order to determine whether the assumptions of regression analysis are satisfied, correlation analysis between independent variables was performed. As a result, the absolute value of the correlation coefficient ranged from 0.15 to 0.65, showing that the correlation between variables was <0.70. In the testing for multicollinearity, the variance inflation factor (VIF) value ranged from 1.000 to 2.056 and did not exceed 10 in all variables, indicating that there is no multicollinearity problem. The Durbin–Watson statistic was 1.893, which is a value close to 2.0, indicating that the problem of autocorrelation can be ignored. In this analysis, a total of four models were presented by the stepwise selection method. In Model 1, the variable that has the greatest effect on quality of life when presented alone was applied. In Model 2, two variables were used with the addition of a variable that had a lower impact on quality of life than the one presented in Model 1. In Model 3, three variables were used in the manner described above. Likewise, in Model 4, a total of four variables were entered, and this model showed that the influencing factor which has the greatest impact on quality of life is PWI‐SF, followed by KOSS, BDI, and working area. The explanatory power of each model was 59.4% (*R* ^2^ = .594) for Model 1, 65.4% (*R* ^2^ = .654) for Model 2, 68.5% (*R* ^2^ = .685) for Model 3, and 69.7% (*R* ^2^ = .697) for Model 4. In addition, all regression models were statistically significant (*F* = 300.510, *F* = 195.117, *F* = 149.733, *F* = 118.876, *p *< .001) (Table 4).

**Table 4:** Mental health domain with working area on quality of life multiple regression analysis

Mental health domain with working area	Model 1	Model 2	Model 3	Model 4
	B	B	B	B
PWI‐SF	−0.963***	−0.744***	−0.577***	−0.576***
KOSS		−0.461***	−0.405***	−0.436***
BDI			−0.434***	−0.451***
Working area				−2.682**
(Constant)	92.783	101.027	98.925	101.094
*F*	300.510***	195.117***	149.733***	118.876***
Adjusted *R* ^2^	.594	.654	.685	.697
∆*R* ^2^		2	.032	.013

Abbreviations: B, Unstandardized Coefficients; BDI, Beck Depression Inventory; KOSS, Korean occupational stress scale; PWI‐SF, Psychosocial Well‐being Index—Short Form.

** *p *< .01.

*** *p *< .001.

## 4 DISCUSSION

This study investigated mental health and quality of life in the firefighters in charge of on‐scene operations in the capital area and growth promotion area. The participants of this study were a total of 206 firefighters including 110 firefighters in the capital area and 96 firefighters in the growth promotion area. Mental health domains were assessed using the Beck Depression Inventory (BDI), Psychosocial Well‐being Index—Short Form (PWI‐SF), and Korean occupational stress scale (KOSS), and the tool used to measure quality of life was the World Health Organization Quality of Life Scale (WHOQOL‐BREF). The results of this study are presented and discussed below.

First, the examination of differences between the groups of firefighters in the capital area and in the growth promotion area showed that there were significant between‐group differences in mental health domains. In addition, there was a significant difference in all the subareas of occupational stress except job demand and job security. In quality of life, there was no significant difference between the two groups of firefighters. On the other hand, in all the other domains except the quality of life index, scores were higher in the capital area than in the growth promotion area, and higher scores indicate higher levels of stress or depression. These results support the findings of Lee's (2017) study on life satisfaction in the capital area and noncapital areas mentioned above, which showed that job satisfaction was higher in noncapital areas. In addition, according to the data of Statistics Korea (2016), the number of residents per firefighter is more than three times higher in the capital area than in noncapital areas (Statistics Korea, 2016). These statistical data are consistent with the study results of Saijo et al., (2008) which showed that higher workloads have a negative impact on mental health among firefighters (Saijo et al., 2008). In this regard, in this study, depression, sociopsychological health stress, and occupational stress were found to be higher in the capital area than in the growth promotion area, and this study result was thought to be due to higher workloads in the capital area compared to the growth promotion area and differences in job satisfaction due to the social and economic factors causing regional disparities. Therefore, since these regional differences may affect mental health among firefighters, it is necessary to provide mental health‐related services suitable for the regional characteristics of the capital area and growth promotion area.

Second, the correlation analysis among mental health domains, quality of life, and working area showed that all mental health domains were correlated with each other. In addition, quality of life was found to have a statistically significant negative correlation with all the domains of mental health. Moreover, there was no direct correlation between working area and quality of life, but they were negatively correlated with mental health domains. These results suggest that the management of mental health should be considered for the improvement of quality of life. Lee et al. (2009) reported that there was a significant correlation between occupational stress and quality of life (Lee et al., 2009). In addition, Lee and Jung (2018) reported that there was a significant correlation between the degree of coping with psychological stress and quality of life (Lee & Jung, 2018). These findings support the results of this study in that they showed a significant correlation between mental health factors and quality of life. However, this study is differentiated from previous studies in that regional differences were considered in this study. The results of this study suggest that sufficient consideration of regional differences is required in addition to the management of sociopsychological health stress including depression and occupational stress in order to improve the level of quality of life among firefighters.

Third, multiple regression analysis was conducted to examine the effects of mental health domains and working area on quality of life, and four models were presented. In Model 1, sociopsychological health stress was presented as the single variable which has the greatest effect on quality of life. In Model 2, occupational stress was entered in addition to the variable of Model 1, and in Model 3, depression was added to the variables of Model 2. In Model 4, the regional difference was entered in addition to the variables of Model 3. These results suggest that mental health factors, such as sociopsychological health stress, occupational stress, and depression, affect quality of life and that quality of life can be improved by the management or interventions for mental health improvement. In addition, such management and interventions are expected to lead to greater improvement in quality of life among the firefighters of the capital area. These results were statistically confirmed by a very large explanatory power of 69% in Model 4, and they place greater emphasis on the need for management or interventions for mental health improvement. Lee et al. (2009) reported that the management of occupational stress to reduce it would help to enhance quality of life in firefighters (Lee et al., 2009). According to Yoon and Kim (2014), depression and low social support are indirect factors affecting occupational stress and thus act as factors lowering quality of life (Yoon & Kim, 2014). However, in this study, the results of regression analysis showed that these factors are all important factors that directly affect quality of life. In addition, the regional difference between the capital area and the growth promotion area was identified as an important factor affecting quality of life. Therefore, direct interventions for mental health factors such as sociopsychological health stress, occupational stress, and depression are crucially required in improving quality of life in firefighters working on the scene. In addition, it is expected that if such interventions are centered on the capital area rather than the growth promotion area, they will lead to greater improvement in quality of life.

The results of this study showed that there was a statistically significant difference in mental health between firefighters in the capital area and those in the growth promotion area. In addition, mental health factors directly affecting quality of life in firefighters were identified, and differences according to the working area were found. First, firefighters in the capital area showed a relatively higher level of depression than those in the growth promotion area and were found to be under sociopsychological health stress and occupational stress. Second, in order to improve quality of life among firefighters working on the scene, there is a need for direct mental health management regarding sociopsychological health stress, occupational stress, and depression taking into account the differences according to the working area.

### 4.1 Limitations

This study has some limitations which need to be pointed out. First, regarding the growth promotion area group, this study was conducted only in some areas across Gangwon and Gyeongbuk Provinces. Therefore, in future research, it is necessary to compare regional differences by expanding the study area to more diverse regions among the growth promotion areas including noncapital areas. Second, causal analysis and factor analysis for the mental health domains which showed differences between groups were not sufficiently carried out. Therefore, in future research, it is necessary to use various factors affecting mental health, and to conduct comparative studies by closely examining regional differences. Third, individual factors were not sufficiently considered. Therefore, in future studies, in order to enhance the reliability of research and carry out various analyses, it is necessary to take into account more individual factors such as frequent on‐scene operations and injury experiences.

## 5 CONCLUSION

The aim of this study was to investigate the effect of mental health on quality of life between the groups of firefighters working in the capital area and those working in the growth promotion area. For this purpose, a survey was conducted from September to November 2018 among a total of 206 participants, including 110 firefighters in the capital area and 96 firefighters in the growth promotion area, and the collected data were analyzed.

The results of this study showed that there were differences in mental health domains among firefighters according to the working area. In addition, among the factors affecting the level of An Analysis of Factors Affecting the Job Stress of Firefighters quality of life among firefighters, sociopsychological health stress was shown to have the greatest impact on it, followed by occupational stress, depression, and the difference in the working area, and they were shown to have the explanatory power of 69%. Therefore, continuous research and analysis are needed on the direct influences of mental health domains such as sociopsychological health stress, occupational stress, and depression on quality of life. In addition, it is considered that an approach considering regional characteristics is needed to implement mental health interventions for firefighters according to the working area.

This study is the first attempt to consider and compare regional differences in the mental health of firefighters in Korea. In addition, it is a significant outcome of this study that with respect to mental health domains, the difference in the working area was found to have a close relationship with the improvement of quality of life.

## CONFLICT OF INTEREST

No conflicts of interest have been reported by the authors or by any individuals in control of the content of this article.

## Data Availability

Research data are not shared.

## APPENDIX 1

### Growth promotion area notice in South Korea

**Table:**

Province	Growth promotion area
Gangwon‐do	Samcheok‐si, Taebaek‐si, Yangyang‐gun, Yeongwol‐gun, Jeongseon‐gun, Pyeongchang‐gun, Hongcheon‐gun, Hoengseong‐gun
Chungcheongbuk‐do	Goesan‐gun, Danyang‐gun, Boeun‐gun, Yeongdong‐gun, Okcheon‐gun
Chungcheongnam‐do	Gongju‐si, Geumsan‐gun, Buyeo‐gun, Seocheon‐gun, Yesan‐gun, Cheongyang‐gun
Jeollabuk‐do	Gimje‐si, Namwon‐si, Jeongeup‐si, Gochang‐gun, Muju‐gun, Buan‐gun, Sunchang‐gun, Imsil‐gun, Jangsu‐gun, Jinan‐gun
Jeollanam‐do	Gangjin‐gun, Goheung‐gun, Gokseong‐gun, Gurye‐gun, Damyang‐gun, Boseong‐gun, Sinan‐gun, Yeonggwang‐gun, Yeongam‐gun, Wando‐gun, Jangseong‐gun, Jangheung‐gun, Jindo‐gun, Hampyeong‐gun, Haenam‐gun, Hwasun‐gun
Gyeongsangbuk‐do	Mungyeong‐si, Sangju‐si, Andong‐si, Yeongju‐si, Yeongcheon‐si, Goryeong‐gun, Gunwi‐gun, Bonghwa‐gun, Seongju‐gun, Yeongdeok‐gun, Yeongyang‐gun, Ulleung‐gun, Uljin‐gun, Uiseong‐gun, Cheongdo‐gun, Cheongsong‐gun
Gyeongsangnam‐do	Miryang‐si, Geochang‐gun, Goseong‐gun, Namhae‐gun, Sancheong‐gun, Uiryeong‐gun, Hadong‐gun, Hamyang‐gun, Hapcheon‐gun
Total	70
